# Tumor‐associated macrophages regulate the function of cytotoxic T lymphocyte through PD‐1/PD‐L1 pathway in multiple myeloma

**DOI:** 10.1002/cam4.4814

**Published:** 2022-05-20

**Authors:** Jiangbo Zhang, Zhaoyun Liu, Panpan Cao, Hao Wang, Hui Liu, Luoming Hua, Hua Xue, Rong Fu

**Affiliations:** ^1^ Department of Hematology Tianjin Medical University General Hospital Tianjin People's Republic of China; ^2^ Department of Hematology Hebei University Affiliated Hospital Baoding People's Republic of China

**Keywords:** CD8 + T cells, CSF1R, multiple myeloma, PD‐1/PD‐L1, TAMs

## Abstract

**Background:**

Tumor‐associated macrophages (TAMs) are originated from circulating mononuclear cells in peripheral blood. They result from the recruitment of tumor cells and are a vital constituent of the tumor microenvironment. TAMs may be involved in the immunological escape of vicious clonal plasma cells (PC) in the bone marrow (BM) of sufferers with myeloma.

**Methods:**

From March 2020 to January 2021, 28 healthy controls (HC) and 86 multiple myeloma (MM) (53 newly diagnosed MM [NDMM] and 33 remissions) patients were enrolled as objects of the study. The expression of TAMs in the BM, CSF1 on CD138 + cells, and CSF1R on macrophages were detected by the method of flow cytometry, and the expression of PD‐1 on CD8 + T cells and PD‐L1 on TAMs were also done. Bone marrow mononuclear cells (BMMNCs) were extracted and cultured into TAMs, CD8 + T cells were sorted by magnetic beads and cultured, a coculture system was established and different inhibitors were added. The expression of the perforin and granzyme B was detected by flow cytometry.

**Results:**

The percentage of TAMs in NDMM group (61.49 ± 2.176%) increased when compared with remission (23.08 ± 1.699%, *p* < 0.001) and HC group (17.95 ± 1.865%, *p* < 0.001), and TAMs decreased after adding CSF1R inhibitor. Moreover, the expression of CSF1 on CD138 + cells increased significantly in NDMM group (17.090 ± 0.9156%) than remission (8.214 ± 0.5911% *p* < 0.001), and HC group (5.257 ± 0.6231%, *p* < 0.001), and CSF1R on macrophages increased significantly in NDMM group (58.78 ± 2.286%) than remission (20.74 ± 1.376%, *p* < 0.001) and HC group (17.42 ± 1.081%, *p* < 0.001). The expression of PD‐1 on CD8 + T cells in NDMM group (32.64 ± 2.982%) increased than remission (20.35 ± 2.335% *p* < 0.01) and HC group (17.53 ± 1.349%, *p* < 0.001), and PD‐L1 on TAMs also increased in NDMM group (50.92 ± 2.554%) than remission (20.02 ± 1.893%, *p* < 0.001) and HC group (13.08 ± 1.289%, *p* < 0.001). When CD8 + T cells were cocultured with TAMs, the perforin and granzyme B levels decreased significantly. However, the perforin and granzyme B levels were partly restored after adding CSF1R inhibitor and anti‐PD‐L1 antibody.

**Conclusion:**

Our study shows that TAMs were increased in MM patients which can inhibit the function of cytotoxic T lymphocyte (CTL) through the PD‐1/ PD‐L1 signaling pathway and participate in the occurrence of immune escape of myeloma cells.

## Graphical abstract

Tumor‐associated macrophages(TAMs) are a vital constituent of the tumor microenvironment and involved in the immunological escape of vicious clonal plasma cells of myeloma patients. Our research shows that CSF1R can regulates the production of TAMs while the PD‐1/PD‐L1 pathway regulates the effect of TAMs on cytotoxic T lymphocyte. It may provide a new basis for targeted therapy of multiple myeloma.

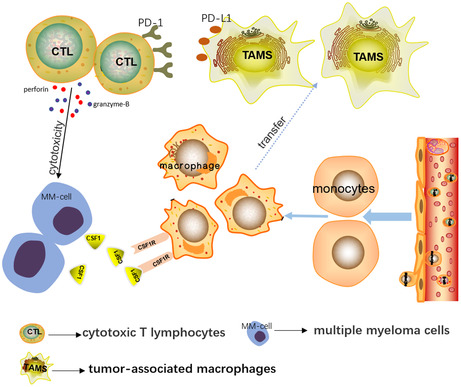

## 1 INTRODUCTION

Multiple myeloma is a malignant hematological tumor, the main pathogenesis is a malignant clonal development of abnormal PC in the BM. The primary clinical characteristics include anemia, excessive bone destruction, kidney failure, hypercalcemia, and infection.^1^ Although proteasome inhibitors, immunomodulators, monoclonal antibodies drugs, medicine for histone deacetylase inhibitors, Bcl‐2 inhibitors, and stem cell transplantation have shown good therapeutic effects, MM still cannot be cured and its occurrence rate is increasing fastly.^2^, ^3^ The high inhomogeneity and drug tolerance of MM lead to a poor prognosis, therefore, new therapies are urgently needed.

Many previous studies have shown that MM cells in the BM microenvironment can directly or indirectly affect their surrounding cells and extracellular matrix components (ECM). This interreaction engenders a huge effect on metastasis and the occurrence of drug resistance in MM cells. Macrophage constitutes a widely dispersed organ system and it plays a vital role in promoting the existence, growth, and transmission of tumor cells.^4^ Previously, macrophages were divided into two different phenotypes based on distinct activation status, the expression of related cytokines, and surface markers, including pro‐inflammatory “M1” and anti‐inflammatory “M2”.^5^ However, binary classification is generally considered inadequate because it is overly simplistic. CD169+ macrophages, TCR+ macrophages, and TAMs were discovered later.^6^

TAMs are microenvironments infiltrating immune cells, which can facilitate the occurrence and development of tumors. The main mechanism is restraining the antitumor immune reaction of cytotoxic T lymphocytes, and meantime boosting the formation of tumor angiogenesis, even accelerating the deterioration.^7^ CSF1 [colony stimulating factor 1 (macrophage)] is considered a typical cytokine to promote tumor development. It can recruit macrophages to tumor sites and assist tumor cells growth by combining with the factor‐1 receptor (CSF1R) on the cell surface.^8^

High expression of PD‐L1 on the surface of tumor cells enables it to escape the immune attack, which results in disease progression. PC from MM patients can produce more PD‐L1 than healthy people and monoclonal gammopathy of undetermined significance (MGUS), especially in relapsed/refractory MM. And PD‐L1^+^ patients with MGUS and asymptomatic MM tend to show disease progression. PD‐L1 combined with PD‐1 activated the Akt pathway in MM cells, which was closely related to tumor proliferation and drug resistance.^9^, ^10^, ^11^ Those data suggest that PD‐L1 plays a crucial role in the disease progression of MM. Hence, we infer that TAMs might be involved in the regulation of the immune microenvironment of MM. Our research found that the percentage of TAMs in MM patients increased. Furthermore, TAMs could inhibit the function of cytotoxic T cells by the PD‐1/ PD‐L1 signal pathway.

## 2 MATERIALS AND METHODS

### 2.1 Patients

This study enrolled 86 MM patients including 53 NDMM and 33 remission patients (median age: 64.5 years, range:47–84, displayed in Table 1). They were diagnosed according to the MM diagnostic criteria launched by the International Myeloma Working Group (IMWG)^12^ and hospitalized in the ward of Hematology, General Hospital of Tianjin Medical University, from March 2020 to January 2021. The HC group included 16 male and 12 female people at the same stage. The study protocol was approved by the ethics committee, and all participants signed informed consent.

**TABLE 1:** Baseline characteristics of MM patients

Demographic variable	MM (*n* = 86)
Sex, *n*(%)	
Male	43 (50.0%)
Female	43 (50.0%)
Age, *n*(%)	
<65 years	51 (59.3%)
≥65 years	35 (40.7%)
Median (range)	64.5 (47–84)
Hemoglobin (g/dL), *n* (%)	
>10	49 (60.0%)
8.5–10	15 (17.4%)
<8.5	22 (25.6%)
Serum calcium (mmol/L), *n* (%)	
>2.75	7 (8.1%)
≤2.75	79 (91.9%)
Serum creatinine (umol/L), *n* (%)	
≥177	20 (23.3%)
<177	66 (76.7%)
Bone lesions, *n*(%)	
Yes	49 (57.0%)
No	37 (43.0%)
Serum albumin (g/L), *n* (%)	
≥35	41 (47.7%)
<35	45 (52.3%)
Serum beta‐2 microglobulin (mg/L), *n* (%)	
≥5.5	39 (45.4%)
3.5–5.5	10 (11.6%)
<3.5	37 (43.0%)
Serum LDH	
≤the upper limit of normal	74 (86.0%)
>the upper limit of normal	12 (14.0%)
Clonal bone marrow plasma cells, *n* (%)	
<10%	13 (15.1%)
10%–20%	24 (28.0%)
>20%	49 (56.9%)
DS stage, *n* (%)	
I	15 (17.4%)
II	17 (19.8%)
III	54 (62.8%)
ISS stage, *n* (%)	
I	21 (24.4%)
II	21 (24.4%)
III	44 (51.2%)
R‐ISS stage, *n* (%)	
I	21 (24.4%)
II	24 (27.9%)
III	41 (47.7%)
M protein type, *n* (%)	
IgA type	21 (24.4%)
IgG type	42 (48.8%)
Light chain type	17 (19.8%)
Non‐secretory type	6 (7.0%)

LDH, Lactate dehydrogenase; DS stage, Durie‐Salmon Staging System; ISS stage, International Staging System; R‐ISS stage, Revised International Staging System.

### 2.2 Flow cytometric analysis

#### 2.2.1 Detection of TAMs

Flow cytometric gating strategy used CD14 + CD68 + CD163 + CD200R ‐ to identify cells as TAMs.^13^ CD14 + CD68 + cells were determined as macrophages and TAMs were gating from macrophages. First, 200 μl of BM samples were collected from MM patients and HC, respectively. Then, according to standard protocol, the single‐cell suspension was stained with the following antibodies (BD Biosciences) at 4°C for 15 min: anti‐CD14‐PerCP, anti‐CD163‐PE, and anti‐CD200R‐APC antibodies. Hemolysin was used to pyrolyze erythrocytes. Subsequently, the cell membrane was permeated with IntraSure Kit (BD Biosciences) and then cells were incubated with anti‐CD68‐FITC antibody for 30 min and cleaned with PBS. Finally, TAMs were detected by flow cytometry (FCM) (Beckman CytoFLEX). In addition, we confirmed that CSF1R inhibitors could inhibit TAMs formation in NDMM. BMMNCs were extracted from the BM of NDMM patients and then cultured in the incubator(5% CO_2_ and 37°C). Mixing medium (Gibco) containing 15% fetal bovine serum(FBS) (Gibco), 0.1 mg/mL streptomycin (Gibco), 100 U/mL penicillin (Gibco), 50 ng/mL M‐CSF (Miltenyi) was used to induced BMMNCs to TAMs in vitro. One group was supplemented with 0.22 μmol/mL CSF1R inhibitor (Pexidartinib), and the control group was added with the volume of PBS instead of CSF1R inhibitor. After 10 days, TAMs were detected by flow cytometry (FCM) (Beckman CytoFLEX).

#### 2.2.2 Detection of CSF1 on CD138 + cells and CSF1R on macrophages

Collected 200 μl BM specimens from MM patients and HC, respectively. Subsequently, labeling the specimens with anti‐CD138‐Bv421, anti‐CD38‐APC, anti‐CSF1‐APC, anti‐CD14‐PerCP, anti‐CD68‐FITC, and anti‐CSF1R‐Bv421 antibodies was done (BD Biosciences). Among these, CD68 is an intracellular marker. The marker of intracellular staining was fixed and permeated with IntraSure Kit (BD Biosciences). Eventually, the data of CSF1 and CSF1R were acquired by flow cytometry (FCM) (Beckman CytoFLEX).

#### 2.2.3 Detection of expression of PD‐1 on CD8 + T cells and PD‐L1 on TAMs

Collected 200 μl BM specimens from MM patients and HC, respectively. Subsequently, labeling the specimens with anti‐CD3‐PerCP, anti‐CD8‐FITC, and anti‐PD‐1‐PE antibodies. Meanwhile, another group was labeled with anti‐CD14‐PerCP, anti‐CD68‐FITC, anti‐CD163‐PE, and anti‐CD200R‐APC and anti‐PD‐L1 antibodies (BD Biosciences). Eventually, the expression of PD‐1 on CD8 + T cells and PD‐L1 on TAMs was detected by flow cytometry (FCM) (Beckman CytoFLEX), and the data were analyzed by Cell QuestTMPro 4.0.2 software.

#### 2.2.4 Functional analysis of perforin and granzyme B in CD3 + CD8 + T cells

CD8 + T cells were obtained from the coculture dishes, then incubated with anti‐CD3‐APC and anti‐CD8‐FITC at 4°C for 30 min. The cell membrane was permeated with IntraSure Kit (BD Biosciences). Subsequently, incubated with PE‐conjugated perforin and PE‐conjugated granzyme B for 30 min, respectively. In the end, the expression of perforin and granzyme B in CD8 + T cells were detected by flow cytometry (FCM) (Beckman CytoFLEX).

## 3 CELL CULTURE

### 3.1 Cultured TAMs

BMMNCs from NDMM patients were isolated by density gradient centrifugation. Next, counting the BMMNCs and seeding cells in 6‐well dishes at the density of 1 × 10^6^ cells/cm^2^. Then the cells were cultured in the incubator (5% CO_2_ and 37°C) to induce BMMNCs to TAMs. Culture media were replaced by half every 3 days to remain MM cells. Eventually, BMMNCs were induced to TAMs for approximately 10 days. The morphology of macrophages was adherent cells, generally round, or oval when observed under the microscope. We found that these macrophages decreased significantly after adding the CSF1R inhibitor. After trypsin digestion, the phenotype and purity were analyzed by flow cytometry, and the purity of CD14 + CD68 + CD163 + CD200R ‐ cells was up to 90%.

### 3.2 Cultured CD8+ T cells

BMMNCs of NDMM patients were extracted and then CD8 + T cells were isolated with CD8 magnetic beads (Miltenyi Biotec). The purity of CD8 + T cells can exceed 95% by flow cytometry. Then CD8 + T cells were resuspended in RPMI 1640 medium (Gibco), supplemented with 15% FBS, meanwhile adding costimulatory molecules anti‐CD3CD28 (10 mg/mL, Miltenyi Biotec) to maintain cell growth for 10 days.

### 3.3 Coculture between CD8 + T cells and TAMs

The induced TAMs and isolated CD8 + T cells were cultured together, and then the coculture system was established. The coming, all the cells were divided into five groups according to the component. The five groups were CD8 + T cells alone (group A); CD8 + T cells with TAMs (group B); CD8 + T cells with TAMs and 20 μg/mL anti‐PD‐L1 antibody (IgG1, MCE) (group C); CD8 + T cells with TAMs and 0.22 μmol/mL CSF1R inhibitor (group D); CD8 + T cells with TAMs, 20 μg/mL anti‐PD‐L1 antibody and 0.22 μmol/mL CSF1R inhibitor (group E). After coculturing for 72 h, the function of CD8 + T cells was detected.

## 4 STATISTICAL ANALYSIS

The whole statistical results were analyzed by SPSS version 21.0 software (IBM, Armonk). The mean ± standard deviation (SD) was adopted to clarify the data. The significance of each group was handled by unpaired *t*‐test and one‐way analysis of variance (ANOVA). When the data distribution is abnormal, a nonparametric test is used. Statistical significance was set at two‐sided *p* < 0.05.

## 5 RESULTS

### 5.1 TAMs increases in MM patients, CSF1R inhibitor decreases the production of TAMs by MM cell

**FIGURE 1:**
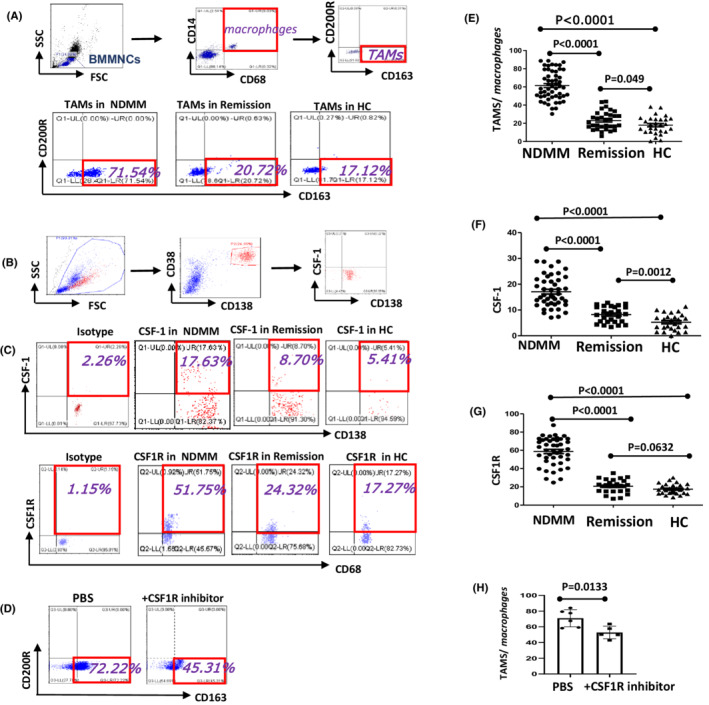
NDMM patients expressed more TAMs and CSF1R inhibitor can decrease the production of TAMs. (A, B, C, D) Representative flow cytometry scatter diagrams of TAMs, CSF‐1, CSF1R in BM of MM and HC groups were shown. (E) The percentage of TAMs in NDMM group was remarkably higher than remission and HC group and the difference between remission and HC group was significant. (F) The expression of CSF1 on CD138 + cells in NDMM group was significantly increased than remission and HC group, and the difference between remission and HC group was significant. (G) The expression of CSF1R on macrophages in NDMM group was higher than remission and HC group, while there was no significant difference between remission and HC group. (H) The percentage of TAMs in NDMM group was decreased after adding CSF1R inhibitor. All the data were shown as mean ± SD. *p* values were obtained by using unpaired *t*‐test and *p*<0.05 was considered significant

CD14 + CD68 + cells were determined macrophages. TAMs (CD14 + CD68 + CD163 + CD200R ‐ cells) were gated from macrophages (CD14 + CD68 + cells) (Figure 1A). The percentage of TAMs in NDMM group (61.49 ± 2.176%) were remarkably higher than that remission (23.08 ± 1.699%, *p* < 0.001) and HC group (17.95 ± 1.865%, *p* < 0.001), and there was an observable distinct between remission and HC group (*p* < 0.05) (Figure 1E). Next, we detected the influencing molecules of TAMs. The expression of CSF1 on CD138 + cells in NDMM group (17.09 ± 0.9156%) significantly increased than remission (8.214 ± 0.5911%, *p* < 0.001) and HC group (5.257 ± 0.6231%, *p* < 0.001), and there was an observable distinct between remission and HC group (*p* < 0.01) (Figure 1B, C, F). Furthermore, the expression of CSF1R on macrophages in NDMM group (58.78 ± 2.286%) was higher than remission (20.74 ± 1.376%, *p* < 0.001) and HC group (17.42 ± 1.081%, *p* < 0.001), while there was no significant difference between remission and HC group (*p* > 0.05) (Figure 1C, G). The percentage of TAMs in NDMM group decreased after adding CSF1R inhibitor (*p* < 0.05) (Figure 1D, H). Next, MM cell line OPM2 which expresses CSF1 (Figure S1B) was cocultured with BMMNCs from NDMM to demonstrated that TAMs decreased after adding CSF1R inhibitor (Figure S1C, D).

### 5.2 The PD‐1 on CD8 + T cells and PD‐L1 on TAMs increases in MM patients

**FIGURE 2:**
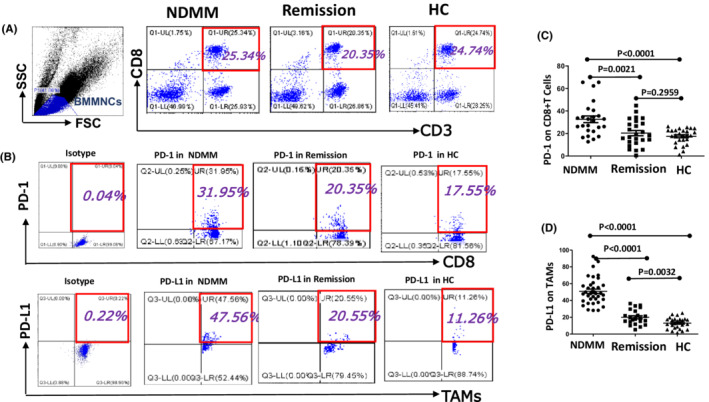
The expression of PD‐1 on CD8 + T cells and PD‐L1 on TAMs in MM and HC groups were detected. (A, B) Representative flow cytometry scatter diagrams of PD‐1 on CD8 + T cells and PD‐L1 on TAMs of MM and HC groups were shown. (C) The expression of PD‐1 in NDMM increased significantly than remission and HC groups, but there was no significant difference between the remission and HC group. (d) The expression of PD‐L1 on TAMs was higher in NDMM group when compared to remission and HC group and there was a significant difference between remission and HC group. All the data were shown as mean ± SD. *p* values were obtained by using unpaired *t*‐test and *p*<0.05 was considered significant

The expression of PD‐1 on CD8 + T cells in NDMM group (32.64 ± 2.982%) was significantly increased than remission (20.35 ± 2.335%, *p* < 0.01) and HC group (17.53 ± 1.349%, *p* < 0.001), there was no significant difference between the remission and HC group (*p* > 0.05) (Figure 2A, B, C). The expression of PD‐L1 on TAMs was higher in NDMM group (50.92 ± 2.554%) when compared with remission (20.02 ± 1.893%, *p* < 0.001), and HC group (13.08 ± 1.289%, *p* < 0.001), and there was significant difference between remission and HC group (*p* < 0.01) (Figure 2B, D).

### 5.3 TAMs restrains the function of CD8 + T cells by PD‐1/PDL‐1 signal pathway

**FIGURE 3:**
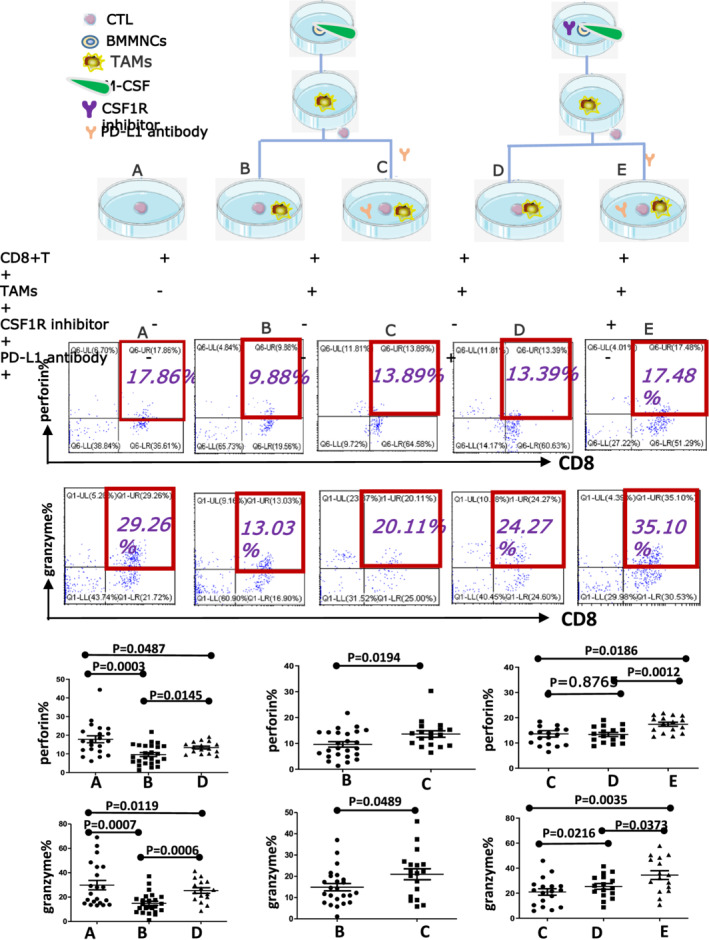
CSF1/CSF1R signaling pathway promotes the formation of TAMs, then TAMs inhibit CTL function through PD‐1/PD‐L1 signaling pathway. The flow chart above shows the expression of perforin and granzyme B in five groups of CD8 + T cells. Statistical analysis showed the functional molecules of CD8 + T cells with TAMs (group B) were lower than CD8 + T cells alone (group A). CTL function was improved after adding CSF1R inhibitor (group D) or anti‐PD‐L1 antibody (group C). And CTL function was improved significantly after adding anti‐PD‐L1 antibody and CSF1R inhibitor together (Group E) and there was a distinction among groups E, C and D. All the data were shown as mean ± SD. *p* values were obtained by using unpaired *t*‐test and *p*<0.05 was considered significant

The functional molecules of CD8 + T cells with TAMs in group B (perforin: 9.622 ± 1.056; granzyme B: 14.91 ± 1.711%) were lower than CD8 + T cells alone in group A (perforin: 17.82 ± 1.887; granzyme B: 29.82 ± 3.882%, *p* < 0.001). Compared group B and D, CTL function in group D (perforin 13.37 ± 0.789; granzyme B: 25.37 ± 2.297%, *p* < 0.05) was improved after adding CSF1R inhibitor. The function of CD8 + T cells improved after adding anti‐PD‐L1 antibody in group C (perforin: 13.61 ± 1.258; granzyme B: 20.97 ± 2.582%) than group B (*p* < 0.05). The CTL function in Group E (perforin: 17.42 ± 0.806; granzyme B: 34.47 ± 3.486%) exceeded the other two groups C (*p* < 0.05) and D (*p* < 0.05) after adding anti‐PD‐L1 antibody and CSF1R inhibitor and there was a distinction between group E and C, D (*p* < 0.05) (Figure 3).

## 6 DISCUSSION

As is known to all that the proliferation of PC in the BM microenvironment of MM is closely related to the disease formation and evolution. Stromal cells and factors bolster the proliferation, survival, homing, immune evasion, and drug tolerance of MM cells. The cell–cell interactions bring about the activation of many signal pathways and finally secret many tumor cytokines. These cytokines include interleukin (IL‐6, IL‐10) which is involved in the survival and growth of MM PC, and vascular endothelial growth factor (VEGF) which can promote angiogenesis. In recent years, several discoveries have highlighted the pleiotropic roles of macrophages, which make up a large proportion of the immune milieu in the BM, in supporting MM disease progression.^14^

Macrophages, which exist in almost tissues, are phagocytic immune cells that exhibit high diversity and plasticity according to their microenvironment.^15^ Previous studies have suggested that many chemokines (CCL5 and CCL2) and cytokines (the VEGF family members and CSF‐1) can be able to recruit circulating inflammatory monocytes to the tumor environment, exerting an enormous effect on the formation of TAMs. While recent studies have found that embryonic‐derived resident macrophages and tissue‐resident macrophages are also very important.^16^, ^17^ Recently, some research also indicated that myeloid‐derived suppressor cells (MDSCs) are another major circulating forebody of TAMs, and C5a is a significant intermediary in the recruitment and TAMs functional polarization.^18^, ^19^ The latest research also found that β_2_‐microglobulin can trigger NLRP3 inflammasome activation in TAMs to boost the progression of MM.^20^ However, there may be many other factors and cells involved, which still need further research. Many histological studies have indicated that macrophages are physically associated with cloned PC in the BM of MGUS and MM. Furthermore, some researchers clearly state that a high expression of total CD68 + macrophages in patients with MM leads to poor results that are unrelated to tumor load or disease stage. In particular, patients with much higher expression of CD68 + macrophages have less complete response rates and worse disease stability and total survival rates, which supports the idea that macrophages may impact MM pathogenesis.^21^, ^22^, ^23^

In our study, we caught sight of a phenomenon that TAMs are highly expressed in NDMM. Furthermore, we analyzed the expression of CSF1 on CD138 + cells as well as the expression of CSF1R on macrophages to find the cause (Figure S1 S1). Eventually, we inferred that the increase in TAMs of MM patients is due to MM cells‐ induced macrophages into TAMs by CSF1/CSF1R.

We know that suppression of CTL in the immunologic system and/or the overexpression of immune checkpoint proteins play a major action in the progression of MM.^24^ Recently, the PD‐1/PD‐L1 axis became an important immune checkpoint in hematological malignancies and solid tumors. All cohort analyses of MM and SMM patients showed CD14 + CD16 + nonclassical monocytes express higher levels of PD‐L1 than classical CD14 + CD16 ‐ cells, and it was unrelated to the disease stage.^25^ Recent studies have indicated that macrophages in MM directly inhibit immune reactions because the tumor microenvironment in MM is deficient to devour tumor cells, display antigens, and activate adaptive immune reactions, which differs from that of macrophages in normal tissues.^26^, ^27^

Our in vitro study showed that PD‐1 on CD8 + T cells and PD‐L1 on TAMs increased. For further research on the interaction between TAMs and CTL cells, we cocultured TAMs and CD8 + T cells, added different inhibitors, and found the changes in functional molecules perforin and granzyme B. It is proved that TAMs play an immunosuppressive role by inhibiting CTL through the PD‐1/PD‐L1 signaling pathway. Now, the most constantly used method to target macrophages is to inhibit the CSF1R receptor.^28^, ^29^ Many ongoing clinical trials have tested humanized monoclonal anti‐CSF1R antibodies and CSF1R inhibitors can be a method of inhibiting TAMs in advanced metastatic solid cancer and hematological malignancies. The preliminary results of these trials showed that the toxicity of CSF1R inhibitors was low and well‐tolerated.^30^, ^31^ Notably, it was determined that CSF1R blockers bound to the chemotherapeutic agents bortezomib or meflanga remarkably decrease tumor load and increase the overall survival in mice with MM tumors, compared to those treated with monotherapy alone.^32^ Some studies have revealed that CSF1 produced by tumor cells can accelerate the accumulation of TAMs, and CSF1R signaling blockade can delay tumor progression and reduced metastasis by depleting TAMs.^33^ Together, these studies emphasized the targeting of macrophages, which suggests that CSF1R inhibitors combined with a standard treatment model are probably a potentially curative treatment for MM. Our experiment confirmed that blocking CSF1R can decrease the percentage of TAMs (Figure 1d, H). Furthermore, we also found that the function of CTL can be restored after decreasing the production of TAMs by blocking CSF1R. Combined blocking CSF1R and PD‐L1 can restore the function of CTL significantly when compared to a single PD‐L1 antibody or CSF1R inhibitor (Figure 3). That means CSF1R regulates the production of TAMs while the PD‐1/PD‐L1 pathway regulates TAMs' effect on CTL.

**FIGURE 4:**
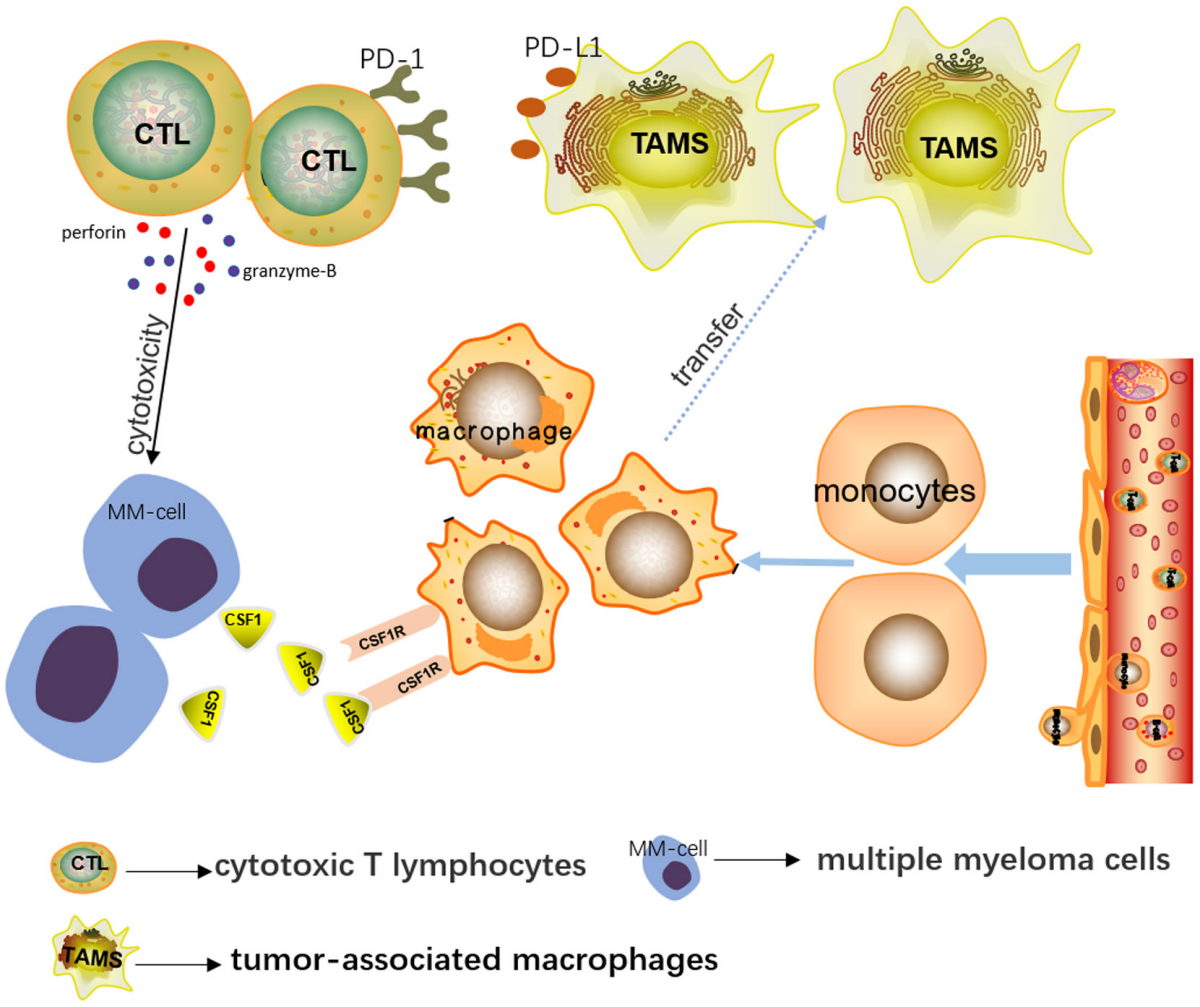
In the bone marrow microenvironment of MM patients, myeloma cells can secrete a large number of CSF1 molecules, recruit peripheral blood circulating monocytes and in situ macrophages to gather around the tumor, and combine with the CSF1R on the surface of macrophages. Under these circumstances, the phenotype and function of macrophages changed, which we called TAMs. High expression of PD‐L1 on TAMs combined with PD‐1 on CTL cells in the tumor microenvironment results in the immune escape of tumor cells. However, when CSF1/CSF1R pathway and PD‐1/PD‐L1 pathway were blocked, the function of effector T cells can be partially or completely restored, providing a new basis for targeted therapy

Therefore, TAMs can exert an important function in the inflammatory response of the tumor microenvironment. For example, they can secrete a variety of cytokines, modulates angiogenesis, inhibit the immune function of T cells, and even promote tumor occurrence, development, metastasis, and deterioration.^34^ CSF1 is a tumor‐promoting cytokine, with the participation of tumor cells, it binds to CSF1R of macrophages, attracts macrophages to the tumor area, and further accelerates the production of TAMs. High expression of PD‐L1 on TAMs combined with PD‐1 on CD8 + T cells in the tumor microenvironment restrains the effect of CTL and participates in MM immune evasion (Figure 4). At present, there are only some preliminary studies on the immune escape of MM, and further studies are still needed to be performed to find a better approach for immunotherapy of MM. Recently study found that extracellular‐regulated protein kinase 5 (ERK5) was necessary for supporting the proliferation of macrophages in tumor transplant. ERK5 can maintain the capacity of macrophages to proliferate by suppressing p21 expression to halt their differentiation program. These studies provided new directions for the treatment of malignant tumors.^35^ Except of CSF1/CSF1R and PD‐1/PDL1 pathway, there are still many other important signaling pathways involved in CTL depletion, such as LAG3, TIGIT, CD96, BTLA, VISTA, TIM‐3, and so on. The role of these pathways in CTL deserves further mechanistic and therapeutic investigations.^36^

There are also some potential limitations in our study. First, CD14 + CD68 + CD163 + CD200R––as the markers of TAM may be a little old as a result of some new specific TAMs markers were found in the latest research, such as TREM2. It will be more convinced if these typical markers were used in this study.^37^ In addition, the results were not representative of what was going on inside the body because these data were obtained only from in vitro experiments. Further work is still needed to investigate the effects of TAMs on CTL in vivo.

## CONFLICT OF INTEREST

All authors announce that they have no conflict of interest.

## AUTHOR CONTRIBUTIONS

JZ and RF designed the study. JZ, ZL, and PC launched the experimental analysis and wrote the manuscript. HW, HL, LH, and HX arranged the data in order and helped to collect and analyze the data. RF, JZ, ZL, and PC made strict revisions to the manuscript and analyzed the data carefully to ensure the correctness of the result analysis. All authors contributed to this study and approved the final manuscript. JZ, ZL, and PC made the same contribution to this study.

## Ethics approval and consent to participate

The use of follow‐up data for this study has been approved by the Ethics Committee of Tianjin Medical University General Hospital. Written informed consent to participate was obtained from every patient.

## Supporting information


Figure S1

## Data Availability

The data that support the findings of this study are available from the corresponding author upon reasonable request.
