# Supplementary material for: Tumor‐associated macrophages regulate the function of cytotoxic T lymphocyte through PD‐1/PD‐L1 pathway in multiple myeloma
Source: Cancer Med. 2022 May 20;11(24):4838–48. doi: 10.1002/cam4.4814 (PMC9761071; doi:10.1002/cam4.4814)
Supplement: Supplementary file 1 — Figure S1 [file CAM4-11-4838-s001.docx]

# Tumor-associated macrophages regulate the function of cytotoxic T lymphocyte through PD-1/PD-L1 pathway in multiple myeloma

**Supporting information**

**1.1** **Detection of CD138+ cells**

We detect CD138+ cells of bone marrow mononuclear cells (BMMNCs) in NDMM, healthy control, and remission groups. BMMNCs were extracted by density gradient centrifugation, then labeled with anti-CD138-APC, and the proportion of CD138 + cells was detected by flow cytometry (Beckman CytoFLEX).

**1.2 Detection of CSF1 on OPM2 cell line**

First, myeloma cell lines OPM2 were incubated with anti-CSF1-APC for 15 minutes in the dark, then used 1ml phosphate buffer salt (PBS) to wash the cells by centrifuging at 1500 rpm, subsequently, removed the supernatant and added 300 µl PBS to each tube to resuspend cells. Eventually, the expression of CSF1 on OPM2 cell line was detected by flow cytometry (Beckman CytoFLEX).

**1.3 Co-culture between BMMNCs and OPM2 cell line**

To detect the percentage of TAMs in NDMM after adding CSF1R inhibitor when BMMNCs were co**-**cultured with OPM2 cell line. OPM2 is a cell of multiple myeloma. First, we isolated BMMNC cells from the bone marrow of NDMM patients, then co**-**cultured BMMNCs with OPM2 cell line. They were co**-**cultured in the incubator( 5% CO_2_ and 37 °C). Mixing medium (Gibco, Germany) containing 15% fetal bovine serum(FBS) (Gibco, Germany), 0.1mg/mL streptomycin (Gibco), 100U/mL penicillin (Gibco), 50ng/ml M-CSF (Miltenyi, Germany) was used to induced BMMNCs to TAMs in vitro. One group was supplemented with 0.22umol/ml CSF1R inhibitor (Pexidartinib), and the control group was added with the volume of PBS instead of CSF1R inhibitor. After 10 days, TAMs were detected by flow cytometry (FCM) (Beckman CytoFLEX).

**Figure legends**

**Figure S1** The percentage of TAMs was decreased after adding CSF1R when co**-**cultured BMMNCs with OPM2 cell line. (a) CD138+ cells existed in NDMM, HC and remission groups. The percentage of CD138+ cells in NDMM is higher than HC and remission groups. (b) CSF1 is expressed on OPM2 cell line. (c, d)When Co-culture BMMNCs and OPM2 cell line, the percentage of TAMs was decreased after adding CSF1R inhibitor.

**Figure legends**


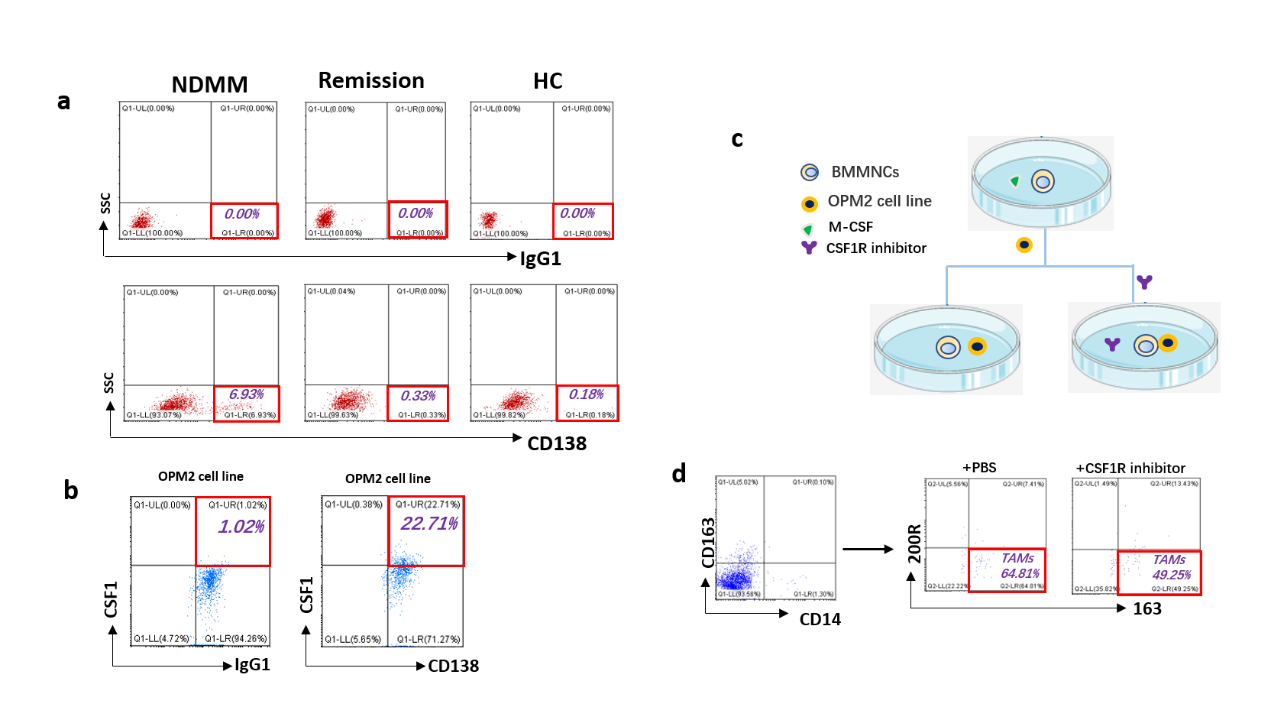


**Figure S1** The percentage of TAMs was decreased after adding CSF1R when co**-**cultured BMMNCs with OPM2 cell line. (a) CD138+ cells existed in NDMM, HC and remission groups. The percentage of CD138+ cells in NDMM is higher than HC and remission groups. (b) CSF1 is expressed on OPM2 cell line. (c, d)When Co-culture BMMNCs and OPM2 cell line, the percentage of TAMs was decreased after adding CSF1R inhibitor.
